# A prospective feasibility study of one-year administration of adjuvant S-1 therapy for resected biliary tract cancer in a multi-institutional trial (Tokyo Study Group for Biliary Cancer: TOSBIC01)

**DOI:** 10.1186/s12885-020-07185-6

**Published:** 2020-07-23

**Authors:** Osamu Itano, Yusuke Takemura, Norihiro Kishida, Eiji Tamagawa, Hiroharu Shinozaki, Ken Ikeda, Hidejiro Urakami, Shigenori Ei, Shigeo Hayatsu, Keiichi Suzuki, Tadayuki Sakuragawa, Masatsugu Ishii, Masaya Shito, Koichi Aiura, Hiroto Fujisaki, Kiminori Takano, Junichi Matsui, Takuya Minagawa, Masahiro Shinoda, Minoru Kitago, Yuta Abe, Hiroshi Yagi, Go Oshima, Shutaro Hori, Yuko Kitagawa

**Affiliations:** 1 Department of Surgery, Keio University School of Medicine, Tokyo, Japan; 2 Department of Hepato-Biliary-Pancreatic and Gastrointestinal Surgery, International University of Health and Welfare School of Medicine, 4-3, Kozunomori, Narita-shi, Chiba, 286-8686 Japan; 3 Department of Surgery, Japanese Red Cross Ashikaga Hospital, Tochigi, Japan; 4 Department of Surgery, Machida Keisen Hospital, Tokyo, Japan; 5 Department of Surgery, Saiseikai Utsunomiya Hospital, Tochigi, Japan; 6 Department of Surgery, Sano Kousei General Hospital, Tochigi, Japan; 7 Department of Surgery, National Hospital Organization Tokyo Medical Center, Tokyo, Japan; 8 Department of Surgery, Eiju General Hospital, Tokyo, Japan; 9 Department of Surgery, National Hospital Organization Saitama National Hospital, Saitama, Japan; 10 Department of Surgery, National Hospital Organization Tochigi Medical Center, Tochigi, Japan; 11 Department of Surgery, Tama Kyuryo Hospital, Tokyo, Japan; 12 Department of Surgery, Fussa Hospital, Tokyo, Japan; 13 Department of Surgery, Kawasaki Municipal Kawasaki Hospital, Kanagawa, Japan; 14 Department of Surgery, Hiratsuka City Hospital, Kanagawa, Japan; 15 Department of Surgery, Tokyo Dental College Ichikawa General Hospital, Chiba, Japan; 16 Department of Surgery, Saitama City Hospital, Saitama, Japan

**Keywords:** Biliary tract cancer, Adjuvant chemotherapy, 1-year administration of S-1, Feasibility study

## Abstract

**Background:**

Although surgery is the definitive curative treatment for biliary tract cancer (BTC), outcomes after surgery alone have not been satisfactory. Adjuvant therapy with S-1 may improve survival in patients with BTC. This study examined the safety and efficacy of 1 year adjuvant S-1 therapy for BTC in a multi-institutional trial.

**Methods:**

The inclusion criteria were as follows: histologically proven BTC, Eastern Cooperative Oncology Group (ECOG) performance status 0 or 1, R0 or R1 surgery performed, cancer classified as Stage IB to III. Within 10 weeks post-surgery, a 42-day cycle of treatment with S-1 (80 mg/m^2^/day orally twice daily on days 1–28 of each cycle) was initiated and continued up to 1 year post surgery. The primary endpoint was adjuvant therapy completion rate. The secondary endpoints were toxicities, disease-free survival (DFS), and overall survival (OS).

**Results:**

Forty-six patients met the inclusion criteria of whom 19 had extrahepatic cholangiocarcinoma, 10 had gallbladder carcinoma, 9 had ampullary carcinoma, and 8 had intrahepatic cholangiocarcinoma. Overall, 25 patients completed adjuvant chemotherapy, with a 54.3% completion rate while the completion rate without recurrence during the 1 year administration was 62.5%. Seven patients (15%) experienced adverse events (grade 3/4). The median number of courses administered was 7.5. Thirteen patients needed dose reduction or temporary therapy withdrawal. OS and DFS rates at 1/2 years were 91.2/80.0% and 84.3/77.2%, respectively. Among patients who were administered more than 3 courses of S-1, only one patient discontinued because of adverse events.

**Conclusions:**

One-year administration of adjuvant S-1 therapy for resected BTC was feasible and may be a promising treatment for those with resected BTC. Now, a randomized trial to determine the optimal duration of S-1 is ongoing.

**Trial registration:**

UMIN-CTR, UMIN000009029. Registered 5 October 2012-Retrospectively registered, https://upload.umin.ac.jp/cgi-open-bin/ctr_e/ctr_view.cgi?recptno=R000009347

## Background

Biliary tract cancer (BTC) includes intrahepatic cholangiocarcinoma, perihilar cholangiocarcinoma, distal cholangiocarcinoma, gallbladder carcinoma, and ampulla of Vater carcinoma. BTC is well-known as one of the most dismal prognostic malignant diseases and its incidence has been increasing [1–3]. Although surgical resection may provide curative treatment, the risk of recurrence is quite high and the reported prognosis of patients with resected advanced BTC is relatively low [4, 5]. Therefore, development of effective perioperative adjuvant therapy is currently being investigated. A meta-analysis series has shown the potential benefit of adjuvant chemotherapy, especially for patients with node-positive resected biliary tract cancer [6]. Despite the potential benefits, no prior randomized control trial (RCT) proved the positive effect of postoperative adjuvant chemotherapy in patients with BTC [7, 8]. Recently, a RCT assessing a 6-month administration of capecitabine for adjuvant therapy for BTC demonstrated improvements in survival [9]; however, the optimal adjuvant chemotherapy regimen for resected BTC has not yet been standardized.

S-1 is well-known as an oral anticancer drug consisting of tegafur, 5-chloro-2, 4-dihydroxypyridine and potassium oxonate. S-1 has already been established as a standardized adjuvant therapy for patients with gastric and pancreatic cancer [10, 11]. Regarding BTC, a phase II trial evaluating unresectable and recurrent cholangiocarcinoma indicated that S-1 had a 35% response rate, and adverse events were also relatively controlled [12]. One prospective phase II trial comparing the efficacy of 6-month administration of S-1 and gemcitabine for adjuvant therapy after curative resection of BTC also showed better prognosis in the S-1 group [13]. Moreover, in Japan, the efficacy of 6-month administration of S-1 for postoperative BTC is currently being investigated in the large-scale phase III ASCOT trial [14]. Thus, S-1 is expected to become a standard treatment in adjuvant therapy for resected BTC.

However, the duration of administration was not verified. One non-inferiority study comparing 1-year administration of S-1 with 6-month administration of S-1 for adjuvant therapy of resected gastric cancer was performed; eventually the study was censored because the 1-year administration group had significantly better prognosis in the interim analysis [15]. 1-year administration is still the standard for the treatment of gastric cancer. Therefore, we hypothesized that 1-year administration of S-1 would improve the prognosis, more than 6-month administration for resected BTC. Although the pilot ASCOT trial showed a high completion rate (75.8%) with 6-month administration of S-1 for BTC adjuvant therapy [16], there has been no conclusive evidence on the feasibility of 1-year administration of S-1. Thus, we planned a phase 2 study to investigate the feasibility of 1-year administration of S-1.

## Methods

### Eligibility criteria

Patients who underwent radical surgery for BTC and who were diagnosed pathologically were eligible if they met the following inclusion criteria: those with BTCs classified into either intrahepatic, hilar/perihilar, or extrahepatic cholangiocarcinoma, gallbladder cancer, or ampullary of Vater carcinomas according to the WHO classification 2010 [17]; Moreover, patients were included, if the eligible pathological stage ranged from Stage IB to Stage III according to the 6th edition of the UICC/AJCC staging system [18] without macroscopic residual tumors; if no distant metastases and no peritoneal dissemination was observed; if no prior chemotherapy or radiation for BTC was administered; patients who were able to start chemotherapy within 10 weeks after surgery; age ≥ 20 years; Eastern Cooperative Oncology Group Performance Status (ECOG-PS) 0 or 1; adequate oral intake; adequate bone marrow function (white blood cells ≥3500/mm^3^, neutrophils ≥2000/mm^3^, platelet ≥100,000/mm^3^, hemoglobin ≥9.0 g/dL), adequate liver function [aspartate aminotransferase (AST) ≤100 IU/L (or 150 IU/L under biliary drainage), alanine aminotransferase (ALT) ≤100 IU/L (or 150 IU/L under biliary drainage)] serum total bilirubin ≤2.0 mg/dL (or ≤ 3.0 mg/dL under biliary drainage), adequate renal function [serum creatinine ≤1.2 mg/dL and creatinine clearance or estimated glomerular filtration rate (GFR) by Cockcroft-Gault formula ≥60 mL/min], and serum albumin ≥3.0 g/dL; normal EKG findings within 28 days before registration; and written informed consent.

The exclusion criteria were as follows: previous history of S-1 administration; uncontrollable diarrhea; history of flucytosine, phenytoin, or warfarin potassium treatments; accumulated pleural effusion or ascites; presence of active infection without viral hepatitis; presence of other cancer except carcinoma in situ within 3 years; severe organ dysfunction (such as heart failure, renal failure, liver failure, intestinal paralysis, uncontrollable diabetes mellitus); presence of pulmonary fibrosis or interstitial pneumonitis; presence of severe mental disorder; presence of severe drug allergy; transfusion within 14 days before registration; women who were pregnant or nursing; women who may have been pregnant or were willing/trying to get pregnant; and unsuitable candidates for this study as judged by the physician.

### Study design (single-arm, non-randomized, open, historical control)

This study was designed by the Keio Surgery Research Network (KSRN) and was conducted at the Keio University Hospital. This study was registered with University Hospital Medical Information Network (UMIN) center (unique trial number: UMIN000009029). Patient registration and data management were conducted at an independent center at Keio University School of Medicine. All laboratory tests required to assess eligibility were completed within 28 days before the start of protocol treatment.

### Treatment schedule

S-1 (tegafur, gimeracil, oteracil potassium; Taiho Pharmaceutical, Tokyo, Japan) was administered within 10 weeks after the surgery. An oral dose of 80 mg/m^2^ S-1 was given every day on days 1 to 28 of a 6-week cycle for a year. The total dose was based on the patient’s body surface area as follows: < 1.25 m^2^, 80 mg; 1.25–1.5 m^2^, 100 mg; > 1.5 m^2^, 120 mg. After a-year of chemotherapy, additional chemotherapy was not given unless the patient was diagnosed with recurrence.

The protocol permitted dose modifications and cycle interruptions were as follows: white blood cells < 2000/mm^3^, neutrophils < 1000/mm^3^, platelet < 75,000/mm^3^, hemoglobin < 8.0 g/dL, adequate liver function (AST > 150 IU/L, ALT > 150 IU/L), serum total bilirubin > 3.0 mg/dL, serum creatinine > 1.5 mg/dL, and adverse events associated with gastrointestinal symptom ≥ Grade 3. In cases for which the S-1 dose was reduced, the dose was decreased by 20 mg/body weight while maintaining a minimum dose of 60 mg/body weight, and it was not subsequently increased for any reason. When dose interruptions were prolonged for longer than 4 weeks or if dose reductions below 60 mg/m^2^ were required, the patient was considered for medication discontinuation. Patients had the option to withdraw from the trial or during follow-up at any stage. Furthermore, criteria for treatment discontinuation included factors such as the physician’s decision, recurrence, and development of other cancers.

### Follow up after surgery

Postoperative follow-up CT scanning were performed at 3, 6, 12 months for the first year and every 6 months following that. Tumor marker tests were conducted every 3 months for 2 years.

### Evaluation of toxicity

Toxicity was categorized according to the Common Terminology Criteria for Adverse Events, version 4.0. Toxicity was recorded during treatment continuously.

### Outcomes

The primary outcome was completion rate at 1 year after first administration of S-1. Secondary outcomes included relative dose intensity (RDI), toxicity, overall survival rate, and disease-free survival rate at 2 years, which was defined as the time from registration until the event. RDI was defined as the proportion of actual dose intensity received to the planned dose intensity.

The expected treatment completion rate was set at 50% based on the data of the ACTS-GC trial, of which completion rate was 65.8% [10]. It was expected that the completion rate would be lower after major hepatobiliary and pancreatic surgeries than after gastric cancer surgery due to increased adverse events and recurrence. The sample size was calculated as 43 patients with a 95% confidence interval for the completion rate of treatment within 30%. Therefore, the target number of patients was set to be 50 for possible ineligible patients.

### Statistical analyses

Data are presented as median (range) or number of patients (%). Intergroup comparisons were performed using the Mann-Whitney U test and chi-square test for continuous and categorical variables, respectively. To identify risk factors for early discontinuation (defined as discontinuation within 2 courses), we performed univariate and multivariate logistic regression analyses. Variables with *P* values < 0.10 in the univariate analysis were included in the multivariate logistic regression analysis. *P* < 0.05 was considered statistically significant. The SPSS 25.0 statistical software (SPSS, Inc., Chicago, IL, USA) was used to perform all the statistical calculations.

## Results

### Patient characteristics

Between June 2011 and December 2014, 50 patients were enrolled in this study. A total of 46 patients were eligible; patient characteristics are summarized in Table 1. The median age was 68.5 years (range, 39–84 years). Nineteen (41%) patients had extrahepatic cholangiocarcinoma, 8 (17%) patients had intrahepatic cholangiocarcinoma, 10 (22%) had gallbladder carcinoma and 9 (20%) had ampulla of Vater carcinoma. Surgical procedures consisted of 25 (54%) pancreatoduodenectomies, 6 (13%) hepatectomies without bile duct resection, 6 (13%) hepatectomies with bile duct resection, and 9 (20%) extended cholecystectomies. Forty-three (94%) patients achieved R0 resection and 20 (46%) had regional lymph node metastases.

**Table 1:** Patient characteristics (*n* = 46)

Variables		n (%) or median (range)
Male: Female		28 (61%)/18 (39%)
Age, years		68.5 (39-84)
ECOG-PS	0	39 (85%)
	1	7 (15%)
Primary disease	Extrahepatic	19 (41%)
	Intrahepatic	8 (17%)
	Gallbladder	10 (22%)
	Ampulla of Vater	9 (20%)
Pathologically stage (UICC)	I	10 (22%)
	II	29 (63%)
	III	7 (15%)
Surgical procedure	Pancreatoduodenectomy	25 (54%)
	Hepatectomy (without bile duct resection)	6 (13%)
	Hepatectomy (with bile duct resection)	6 (13%)
	Extended cholecystectomy	9 (20%)
Morbidity (Clavien-Dindo ≥3)	Total	10 (22%)
	Pancreatic fistula	8 (17%)
	Liver abcess	1 (2%)
	Intraabdominal abcess	1 (2%)
Residual tumor	0	43 (94%)
	1	3 (7%)
Lymph node metastasis	Positive	20 (46%)
CEA, ng/dL		1.8 (0.1-54.0)
CA19–9, ng/dL		17.0 (1.0-3197)

*Abbreviations*: *ECOG-PS* Eastern Cooperative Oncology Group Performance Status, *CEA* carcinoembryonic antigen, *CA19–9* carbohydrate antigen 19–9

### Feasibility analysis (Tables 2, 3, Supplementary Table 1)

Table 2 shows the main results. The completion rate for all patients was 54.3% while the completion rate without recurrence during the 1 year administration was 62.5%. The median relative dose intensity was 62.9%. Of 25 patients with completion, 13 needed dose reduction or temporary therapy withdrawal, 13 patients withdrew from S-1 administration owing to adverse events and 8 of these discontinued cases were due to gastrointestinal adverse events. The reason for discontinuation is summarized in Table 3. Nine cases discontinued because of adverse events at the first course and 3 cases discontinued at the second course. Only one case withdrew after receiving 2 courses due to adverse events. We analyzed the risk factors for early discontinuation, which was defined as discontinuation within 2 courses due to adverse events (Supplementary Table 1). We divided the patients into two groups: the early discontinuation group (*n* = 12) and the remaining patients (*n* = 34). Multivariate analysis identified the age of patients (≥ 69 years old) as an independent risk factor of early discontinuation (HR: 6.5, 95% confidence interval (CI): 1.2–40.0, *P* = 0.03).

**Table 2:** Main outcomes

	n (%) or median (range)
Days from operation to administration, day	54 (31–70)
Completion rate, %	25 (54.3%)
Completion rate without recurrence, %	25 (62.5%)
Reson of cessation (*n* = 21)	
Recurrence	6 (28.6%)
Adverse event	13 (61.9%)
Gastrointestinal	8
Myelosuppression	2
Stomatitis	1
Cholangitis	1
Chest pain	1
Others	2 (9.5%)
Traffic accident	1
House-moving	1
Relative dose intensity, %	62.9 (0.7–100)

**Table 3:** The reason of discontinuation

Course No.	No. of discontinued patients	Reason of discontinuation		
		Adverse event	Recurrence	Other
1	9	Gastrointestinal, 6 Myelosuppression, 1 Cholangitis, 1 Chest pain, 1	–	–
2	4	Gastrointestinal, 2 Myelosuppression, 1	1	–
3	1	–	1	–
4	2	–	2	–
5	1	–	1	–
6	1	–	–	Traffic accident, 1
7	1	–	–	House-moving, 1
8	2	Stomatitis 1	1	–

### Completion rate by primary disease and surgical procedures (Table 4)

Completion rate for all patients and those without recurrence based on their primary disease and surgical procedures are shown in Table 4. The completion rate excluding recurrent cases ranged from 60.0 to 66.7% by the type of surgical procedures.

**Table 4:** The completion rate by primary disease and surgical procedure

	Primary disease				Total
	Extrahepatic	Intrahepatic	Gallbladder	Ampulla of Vater	
(a) Full analysis set (*n* = 46)					
Pancreatoduodenectomy	6 /15 (40.0%)	–	1/1 (100%)	5/9 (55.6%)	12/25 (48.0%)
Hepatectomy without bile duct resection	–	4/6 (66.7%)	–	–	4/6 (66.7%)
Hepatectomy with bile duct resection	2/4 (50.0%)	1/2 (50.0%)	–	–	3/6 (50.0%)
Extended cholecystectomy	–	–	6/9 (66.7%)	–	6/9 (66.7%)
Total	8/19 (42.1%)	5/8 (62.5%)	7/10 (70.0%)	5/9 (55.6%)	25/46 (54.3%)
(b) Cases excluding recurrent cases (*n* = 40)					
Pancreatoduodenectomy	6/13 (46.2%)	–	1/1 (100%)	5/6 (83.3%)	12/20 (60.0%)
Hepatectomy without bile duct resection	–	4/6 (66.7%)		–	4/6 (66.7%)
Hepatectomy with bile duct resection	2/4 (50.0%)	1/1 (100%)	–	–	3/5 (60.0%)
Extended cholecystectomy	–	–	6/9 (66.7%)	–	6/9 (66.7%)
Total	8/17 (47.1%)	5/7 (71.4%)	7/10 (70.0%)	5/6 (83.3%)	25/40 (62.5%)

### Adverse events (Table 5)

Adverse events are shown in Table 5. In total, 41 (89%) patients suffered adverse events (any grade). Hematological events were most common in all grade adverse events. Overall, 7 (15%) patients suffered severe adverse events at grade 3 or more. Gastrointestinal events such as anorexia or diarrhea were more frequent than hematologic events or other events.

**Table 5:** Adverse events

	Grade1	Grade2	Grade3	Grade4	All grade	Grade ≥ 3
Total	38 (83%)	18 (39%)	7 (15%)	–	41 (89%)	7 (15%)
Hematologic						
Neutropenia	5	4	1	–	10 (22%)	1 (2%)
Leukocytopenia	7	6	–	–	13 (28%)	–
Anemia	13	5	2	–	20 (43%)	2 (4%)
Thrombocytopenia	6	2	–	–	8 (17%)	–
Gastrointestinal						
Nausea	9	2	1	–	13 (26%)	1 (2%)
Vomiting	2	0	1	–	3 (7%)	1 (2%)
Anorexia	5	4	2	–	11 (24%)	2 (4%)
Diarrhea	4	5	2	–	11 (24%)	2 (4%)
Abdominal pain	–	–	1	–	1 (2%)	1 (2%)
Others						
Total bilirubin elevation	4	–	–	–	4 (9%)	–
AST elevation	2	–	–	–	2 (4%)	–
ALT elevation	1	–	–	–	1 (2%)	–
Creatinine elevation	1	1	–	–	2 (4%)	–
Aphthous stomatitis	3	2	–	–	5 (11%)	–
Fatigue	11	7	–	–	18 (39%)	–
Pigmentation	11	–	–	–	11 (24%)	–
Rash	3	3	–	–	6 (13%)	–
Chest pain	–	1	–	–	1 (2%)	–

*Abbreviations*: *AST* aspartate aminotransferase, *ALT* alanine aminotransferase

### Long-term outcome (Fig. 1)

**Fig. 1:**
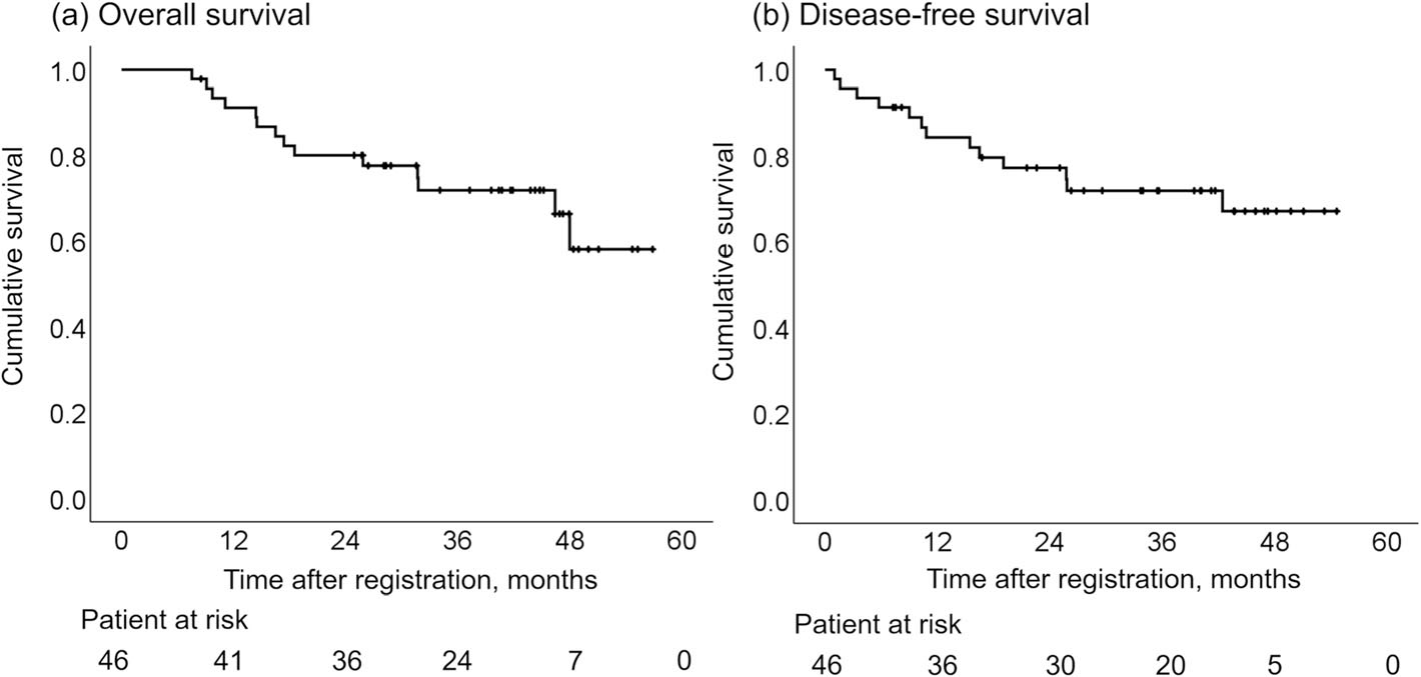
Survival analysis. Kaplan-Meyer curves for overall survival (**a**) and disease-free survival (**b**) are shown

The median follow-up time for all patients in this study was 38.4 months (range, 7.5–56.8 months). The 2-year OS and DFS were 80.0% (95% CI, 68.2–91.8%) and 77.2% (95% CI, 64.7–89.7%) and, respectively (Fig. 1). Eight (60%) of 14 patients who had recurrence in this study period developed recurrence in the liver. The other recurrence sites were as follows: lymph nodes, 5; lung, 3; local recurrence, 2; peritoneal dissemination, 2 and bone, 2.

## Discussion

In this study, we evaluated the feasibility of adjuvant chemotherapy by assessing the outcomes of 1-year administration of S-1 for resected BTC. Our prospective phase II study demonstrated that a completion rate without recurrence during the 1-year administration of S-1 was over 60% and the rate was 50% or more regardless of the surgical procedures or primary disease. The most frequent reason for withdrawal was gastrointestinal adverse events occurring early in the treatment course.

The completion rate in this study was 54.3% (when recurrence cases were excluded, the rate was 62.5%). Previous reports regarding adjuvant chemotherapy for resected gastric cancer showed that 1-year administration of S-1 was tolerable in 48.6–65.8% of patients (in those without recurrence, 60.7–69.1%) [10, 19]. Several studies have evaluated the 6-month administration of S-1 in BTC. One reported the completion rate was 51.4% (the rate for those without recurrence was not available) for BTC after major hepatectomy [13] and the other reported a complete rate of 75.8% (the rate for those without recurrence, 86.0%) [16]. Regarding other types of cancer, a 6-month administration of S-1 was completed in 76.5% of cases (rate for non-recurrence, not available) in colon cancer [20] and 72.2% (rate for those without recurrence, 75.8%) in pancreatic cancer [11]. Compared to other regimens for BTC, the BILCAP trial that evaluated a 6-month administration of capecitabine and the BCAT trial that evaluated a 6-month administration of gemcitabine showed the complete rates were 54.7 and 52.1%, respectively [9, 21]. In the current study, 65.2% (those without recurrence, 70.0%) completed a four-course administration (data were not shown), which seems to be almost acceptable and comparable with other cancers or other regimens.

This study showed a higher incidence of gastrointestinal adverse events compared to that of the phase II trials for unresectable or recurrent BTC [12] and a high incidence of early discontinuation, especially among elderly patients. Specifically, there were several patients who had their medication discontinued due to refusal following grade 1 or 2 gastrointestinal adverse reactions. The abovementioned findings could be attributed to the influence of surgery. Most of the curative surgeries performed for BTC were extremely invasive with extensive lymph node dissections and upper-gastrointestinal reconstructions such as pancreatoduodenectomy or major hepatectomy with extra bile duct resection. Similar data were reported after gastrectomy or major hepatectomy [19] [13]. In a recent study, older age and prescription by surgeons were reported as risk factors for S-1 discontinuation in gastric cancer [22]. In this study, S-1 was administered by surgeons, which might have caused early discontinuation due to insufficient dose modification or medication for adverse events. Another recent prospective study demonstrated that the completion rate of adjuvant therapy increased with combining Kampo for appetite increase [23]. This result showed the importance of control or prevention of gastrointestinal symptoms in patients who have undergone upper abdominal surgery. Therefore, we suggest prophylactic treatment for gastrointestinal symptoms for older patients or prescription by oncologists to avoid early discontinuation. However, it should be noted only one patient discontinued treatment due to a gastrointestinal adverse event after the second course. These results suggest 1-year administration may be tolerable for patients who can receive administration for 6 months.

The ASCOT trial is evaluating the efficacy of 6-month administration of S-1 postoperatively for patients with bile duct cancer [14]. However, the duration was decided according to the adjuvant therapy regimen for pancreatic cancer [11]. There was no evidence regarding the duration of administration. Rather, in a non-inferiority study comparing the 1-year administration of S-1 with a 6-month administration for gastric cancer, the 1-year administration group had better prognosis in the interim analysis. Thus, 1-year administration is still the standard for gastric cancer treatment [15]. Our study showed nearly 80% of 2-year recurrent-free survival. This result seems promising, although this cohort included more than 40% of patients with positive lymph nodes, which is a common poor prognostic factor in BTC as referred to in Japanese registry data or other clinical trials [4, 9, 13, 16]. Because our results about feasibility and prognosis were acceptable, we started a prospective randomized controlled trial in 2018 to evaluate the efficacy of 1-year administration of S-1 as adjuvant chemotherapy by comparing that of 6-months administration of S-1 (TOSBIC-03 trial UMIN: 000029421) for adjuvant therapy of BTC. We are expecting that this study will show a significant survival benefit for 1-year administration with high completion rate and that the 1-year administration of S-1 could be one of the standard treatments after curative surgery for BTC.

## Conclusion

The 1-year administration of adjuvant S-1 therapy for resected BTC was feasible. This regimen has a potential to become a promising treatment for resected BTC.

## Supplementary information

**Additional file 1: Table S1.** Univariate analysis for early discontinuation (within 2 courses)

**Additional file 2.** The list of ethics committees and the reference number

## Data Availability

The protocol and the datasets are available from the corresponding author on reasonable request.

## Abbreviations

BTC: Biliary tract cancer
DFS: Disease-free survival
OS: Overall survival
RCT: Randomized control trial
ECOG-PS: Eastern Cooperative Oncology Group Performance Status
AST: Aspartate aminotransferase
ALT: Alanine aminotransferase
GFR: Glomerular filtration rate
RDI: Relative dose intensity
CI: Confidence interval
CEA: Carcinoembryonic antigen
CA19–9: Carbohydrate antigen 19–9
