# Supplementary material for: A prospective feasibility study of one-year administration of adjuvant S-1 therapy for resected biliary tract cancer in a multi-institutional trial (Tokyo Study Group for Biliary Cancer: TOSBIC01)
Source: BMC Cancer. 2020 Jul 23;20:688. doi: 10.1186/s12885-020-07185-6 (PMC7379785; doi:10.1186/s12885-020-07185-6)
Supplement: Supplementary file 2 — Additional file 2. The list of ethics committees and the reference number [file 12885_2020_7185_MOESM2_ESM.docx]

**Additional file 2. The list of ethics committees and the reference number**

This trial was approved by the ethics committees of Tachikawa hospital (#2013-10), Kawasaki Municipal Ida Hospital (not applicable), Eiju General Hospital (#2011-32), Japanese Red Cross Ashikaga Hospital (not applicable), Saiseikai Utsunomiya Hospital (not applicable), National Hospital Organization Tokyo Medical Center (R12-067), National Hospital Organization Saitama National hospital (R2012-015), Tama Kyuryo Hospital (#23-2), Isehara Kyodo Hospital (#2013-26), Hiratsuka Municipal Hospital (#24-011), Tokyo Dental College Ichikawa General Hospital (#285) and Kawasaki Municipal Kawasaki Hospital (#24-627), and clinical ethics committees of Sano Kousei General Hospital (#24-5), Kitasato Institute Hospital Research Ethics Committee (#12021).
